# Response to Immunotherapy in Sclerosing Epithelioid Fibrosarcoma: Case Report and Literature Review

**DOI:** 10.7759/cureus.50967

**Published:** 2023-12-22

**Authors:** Anna S Koerner, Maggie Zhou, Ashley Brook, Sam S Yoon, Kristen N Ganjoo

**Affiliations:** 1 Surgical Oncology, Surgery, Columbia University Irving Medical Center, New York, USA; 2 Hematology and Medical Oncology, Stanford University School of Medicine, Stanford, USA

**Keywords:** pazopanib, pembrolizumab, nivolumab, ipilimumab, sarcoma, sclerosing epithelioid fibrosarcoma

## Abstract

Sclerosing epithelioid fibrosarcoma (SEF) is an extremely rare subtype of sarcoma that appears histologically low-grade yet usually has a clinically aggressive course with a high rate of local recurrence and distant metastasis. However, these recurrences and metastases often occur years after initial treatment. Metastases can be to the lung as well as extra-pulmonary sites. In this case report, we discuss a patient who developed SEF in the deep soft tissue with metastases. This patient underwent checkpoint inhibitor therapy, with disease response. Thus, SEF is a sarcoma subtype with a unique tumor biology, and immunotherapy may be a promising avenue for treatment.

## Introduction

There are over 100 different subtypes of sarcoma, and these subtypes display a wide variety of clinical behaviors [1]. Sclerosing epithelioid fibrosarcoma (SEF) is a rare subtype of sarcoma typically found in the deep tissues of the extremities. Based on the usual criteria for grading (i.e., mitotic rate, pleomorphism, necrosis), SEF usually appears histologically low grade [2]. There are about 200 SEF cases reported, with only eight articles including clinical outcomes of more than ten patients [3-9]. In these eight articles, the local recurrence rate ranged from 25 to 100%, and the distant metastasis rate ranged from 43 to 90%. The median time to local recurrences ranged from 23 to 57 months, and the median time to distant metastasis ranged from 11 to 92 months. Here, we present a case of SEF that began in deep soft tissues and metastasized with response to immunotherapy and radiotherapy.

## Case presentation

**Figure 1:**
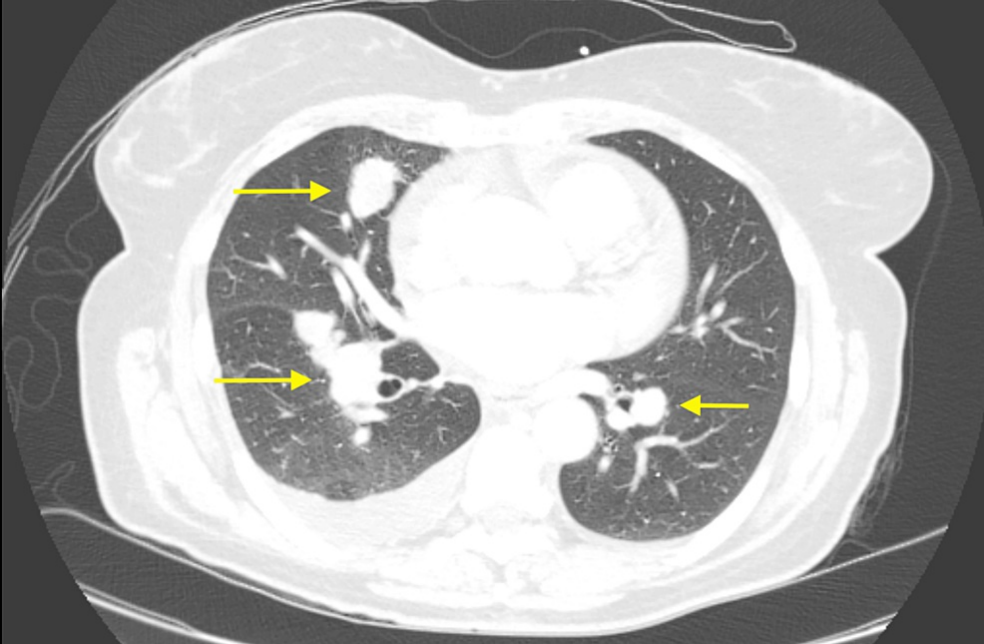
December 2022 CT chest with multiple mass-like consolidations. The yellow arrows point to the consolidations.

**Figure 2:**
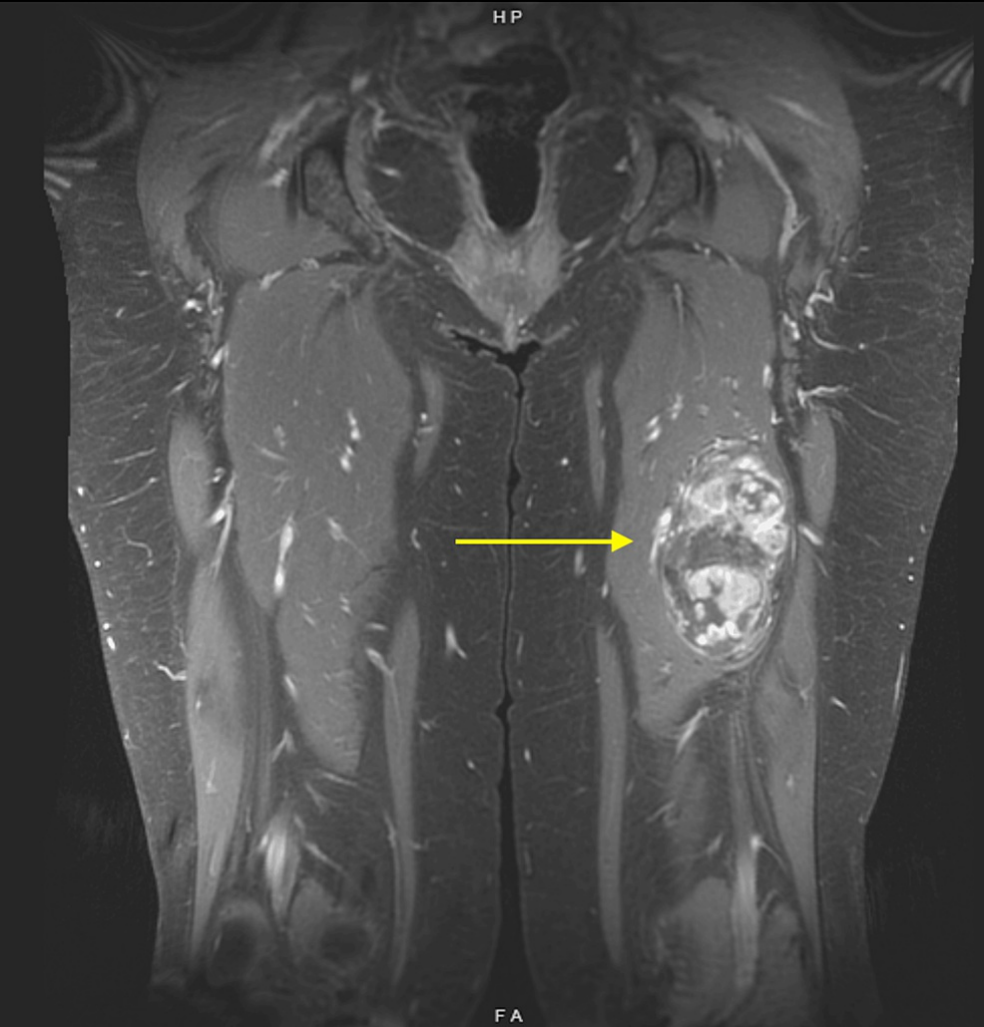
May 2023 MRI of left thigh primary tumor. The yellow arrow points at the tumor.

The patient is a 55-year-old woman who initially developed shortness of breath, cough, and right-sided pleuritic chest pain in August 2022. She was hospitalized for four days in December 2022 after developing flu-like symptoms, at which time a CT chest revealed multiple right upper and lower lung mass-like consolidations, a small right pleural effusion, and ipsilateral intrathoracic lymphadenopathy (Figure 1). She underwent an infectious workup, which was unrevealing, and a CT-guided transthoracic needle biopsy, which showed atypical epithelial cells in a background of inflammation. She was treated empirically for infection with doxycycline without improvement in her symptoms. Subsequent imaging revealed a primary tumor in the left thigh with likely metastases to the lung and mediastinum (Figure 2).

**Figure 3:**
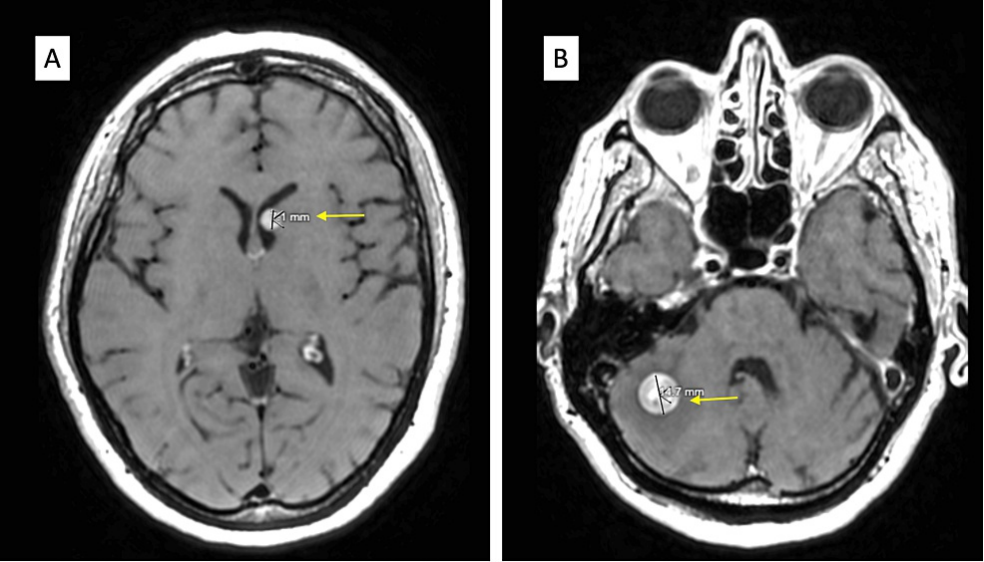
A) May 2023 MRI brain with a yellow arrow pointing to left thalamic metastasis. B) May 2023 MRI brain with a yellow arrow pointing to right cerebellar metastasis.

In March 2023, a right middle lung biopsy showed histological findings consistent with SEF. The thigh mass was not biopsied before resection owing to high clinical suspicion that it was the primary mass on presentation. Next-generation sequencing revealed a low tumor mutation burden (1 mut/Mb), microsatellite stability, and FUS-CREB3L2 fusion. She was started on ifosfamide with concurrent palliative radiotherapy (30 Gy, 10 fx) for superior vena cava compression in April 2023. CT in May 2023 demonstrated two brain metastases involving the left thalamus (Figure 3a) and right cerebellar region (Figure 3b), which were treated with CyberKnife (Accuray Incorporated, Sunnyvale, California) radiation. She was started on ipilimumab, a CTLA-4 inhibitor, and nivolumab, a PD-1 inhibitor, in May 2023 as part of routine clinical care outside of a clinical trial protocol. Immunotherapy was continued for five cycles up to the point of the last clinical follow-up in August 2023. Immunotherapy was briefly paused in June 2023 because of her developing pneumonitis, which resolved with prednisone; she resumed immunotherapy after clinical resolution. There were no other immune-related adverse events.

**Figure 4:**
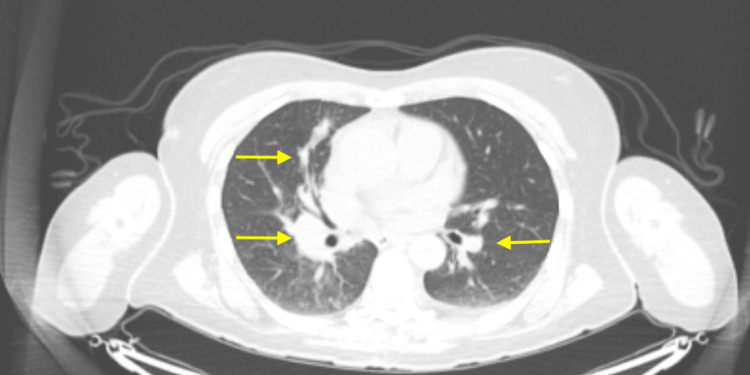
August 2023 CT chest showing response to radiation and immunotherapy. Yellow arrows point to the same mass-like consolidations from Figure 1, which are decreased in size.

**Figure 5:**
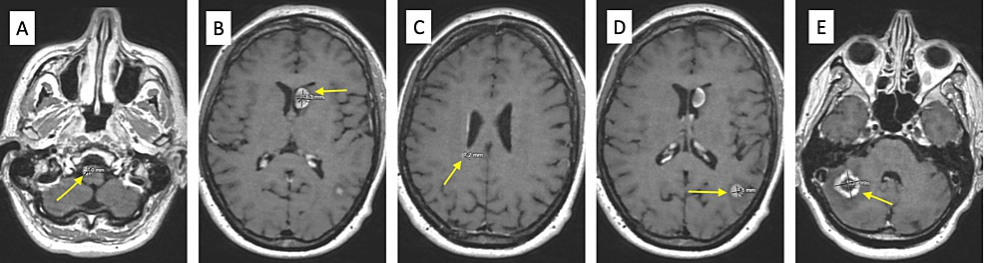
August 2023 MRI brain showing metastases in the A) right medulla, B) left thalamus, C) right periventricular white matter, D) left parieto-occipital region, and E) right cerebellum. Yellow arrows in each figure point to metastases.

During her treatment with immunotherapy, she also underwent neoadjuvant radiation 30 Gy/5 fx to the left thigh, followed by surgical resection of the primary tumor in August 2023. Pathology demonstrated SEF, 10.5 cm, with treatment effect and negative margins. PET/CT showed metastatic thoracic disease responding to immunotherapy (Figure 4), but a new hypermetabolic left inguinal lymph node was suspicious for a new nodal metastasis. MRI brain showed progression of disease of brain metastases (right medulla (Figure 5A), left thalamus (Figure 5B), right periventricular white matter (Figure 5C), left parieto-occipital region (Figure 5d), and right cerebellar region (Figure 5e), which were subsequently treated with CyberKnife radiation, 24 Gy in three fractions to the medullary metastasis, and 18-22 Gy in one fraction to the others. She is alive with the disease, with the last clinical follow-up in August 2023, and plans to continue immunotherapy.

## Discussion

Sarcomas are formally defined as malignant tumors arising from tissues of mesodermal origin [10]. In 2023, it is estimated that there will be 17,370 new cases of sarcoma diagnosed in the United States, accounting for 0.7% of new cancer cases and resulting in 7,280 deaths [11]. There are now more than 100 histologic subtypes of sarcoma as defined by the World Health Organization (WHO) [1]. These histologic subtypes display a wide variety of clinical behavior, and, thus, knowledge of the subtype is important for predicting clinical behavior in individual patients. Sarcomas are also often characterized by histologic grade, which is determined by pathologic features including cellular pleomorphism, necrosis, and mitotic activity [12]. Low-grade tumors can recur locally but do not tend to metastasize, while high-grade tumors can both recur locally and metastasize. The metastasis rate for high-grade tumors often correlates with tumor size.

SEF is a rare subtype of sarcoma. SEF affects patients over a wide range of ages, ranging from childhood to the elderly, with the median age around 45 years old [8,13]. There is roughly equal sex distribution. These tumors commonly arise in the deep soft tissues of the extremities and limb girdles, but SEF can occur in many different locations. It has been described to occur in sites such as the kidney [14], pancreas [15], cecum [16], lung [17], pituitary [18], liver [19], and small intestine [20]. Additionally, a subset of SEF involves the oral and maxillofacial region [21]. Although less common, SEF has been described to originate intraosseously [22-24]. SEF primary tumor size in various series has been shown to range from 3.2 to 29.0 cm, with a median of 8.2 cm [25]. Clinical presentation of SEF is variable and dependent on location.

On CT with IV contrast, tumors often show mild enhancement in the arterial phase with moderate heterogenous enhancement in the portal and late phases [26]. In this case, the patient's lung metastases demonstrated enhancement with contrast and relatively well-defined borders, as expected. MRI images after IV contrast show heterogeneous and/or perilesional enhancement, as seen in the MRI thigh of this case report [27]. On PET, case reports have described standardized uptake values (SUV), ranging from 1.3 to 2.4 [28,29]. Once imaging such as CT and PET scan is performed, biopsy is the next step in diagnosis.

On histology, SEF presents as small- to medium-sized epithelioid-like cells with characteristics of fibroblasts such as cytoplasmic intermediate filaments and well-developed networks of rough endoplasmic reticulum [6,7]. The mitotic range is low, ranging from one to 18 mitoses per 10 hpf with a median of four per 10 hpf [2]. In immunohistochemistry, vimentin, an intermediate filament protein, is a useful marker for the diagnosis of SEF [7]. Prior studies have shown that MUC4, which promotes cell proliferation and represses apoptosis by altering cell adhesive properties, is sensitive and specific for both low-grade fibromyxoid sarcoma (LGFMS) and SEF [30-32].

With fluorescence in situ hybridization (FISH), about 90% of LGFMS cases display a FUS-CREB3L2 gene fusion following translocation t (7;16) [33]. Wang et al. (2020) found that FUS rearrangement is relatively rare in “pure” SEF, although FUS rearrangement was demonstrated in this case [5]. A study by Warmke et al. found that EWSR1-CREB3L1 fusion occurs commonly in SEF cases [25]. There have been a few recent reports of an EWSR1-CREB3L3 fusion being found in SEF, which drives upregulation of the PI3K/mTOR signaling pathway [34-36]. 

The initial differential diagnosis of SEF commonly includes metastatic carcinoma, other epithelioid tumors, Ewing sarcoma, and osteosarcoma. Additionally, SEF shares morphologic, immunohistochemical, and molecular features with low-grade fibromyxoid sarcoma (LGFMS), supporting the thought that these are closely related tumors [37]. However, SEF tends to be more aggressive than LGFMS, has a higher propensity for bone and periosteal involvement, and frequently progresses to abdominal disease. 

There are no definitive guidelines on the optimal therapeutic strategy; however, surgery, radiation, and medical therapy (including chemotherapy and immunotherapy) are the modalities used. The mainstay of treatment for SEF is surgical excision with wide margins whenever possible. However, the microscopic involvement of surgical margins after radical resection is common, indicating that SEF may be more locally infiltrative than is suggested based on imaging [3]. Surgical margins of the primary tumor, in this case, were negative for sarcoma following treatment with radiation and immunotherapy. SEF, unfortunately, has limited response to chemotherapy, although this patient was treated with palliative ifosfamide. A retrospective cohort study of 13 patients with SEF found that response to standard chemotherapy regimens for soft tissue sarcomas (e.g., doxorubicin, ifosfamide with doxorubicin) was limited; disease stabilization occurred in three patients for up to seven months. Median progression-free survival was 2.5 months [3]. Thus, while it may be reasonable to offer palliative chemotherapy to patients with SEF, it will likely only benefit a minority of patients. Adjuvant radiotherapy is frequently used around the time of surgical resection to prevent recurrence and/or slow the progression of disease, although there are limited studies regarding its efficacy. Palliative radiotherapy can also be used to treat sequelae of the SEF mass effect, as in the presented case.

Immunotherapy may be a future avenue for the treatment of SEF. A retrospective review of 88 patients with metastatic soft tissue sarcomas found that treatment with immune checkpoint inhibitors (including pembrolizumab, nivolumab, and ipilimumab) had clinical benefit in 46.8% of patients receiving pembrolizumab and 70.4% receiving combination nivolumab and ipilimumab [38]. In this report, the patient had a favorable response to a combination therapy of nivolumab and ipilimumab. However, this was alongside concurrent radiotherapy, making it difficult to conclusively say if immunotherapy was the driving cause for clinical response. Her clinical response may support the findings of a case series where treatment with ipilimumab and nivolumab was effective in causing regression of metastatic disease [39]. The exact reason for this responsiveness remains unclear. More studies are needed to determine if SEF is immunogenic and characterized by tumor cell PD-L1 expression and high frequencies of infiltrating CD8+ T-cells, which could explain the response to immunotherapy.

Despite its relatively low-grade appearance, SEF generally acts as a highly malignant tumor with local recurrences occurring in over half of patients and distant metastasis occurring in 40%-80% of patients. The main difference between SEF and other highly malignant sarcoma subtypes is that local recurrences and distant metastases often occur years later. In two studies of SEF, local recurrences occurred after a median of 26 months and a broad range of three to 178 months [8,25]. The median time from diagnosis to metastasis is 7.7 years [6]. However, in two separate studies, 17% and 27% of patients presented with metastases at the time of diagnosis, as in this case report [3,8]. It is unknown how long ago the primary thigh tumor in this case report developed before she developed clinical symptoms, which speaks to the frequently slow and indolent course of SEF.

SEF has a propensity to metastasize to the lungs (70%) and bones (41%), though metastases to the liver and brain (as in this case) have also been reported [6,8]. In a study by Ossendorf et al., 38% of patients have metastases in multiple sites [8]. They found that distant disease may be independent of tumor size, with similar rates of metastasis across tumors <5 cm, tumors 5-10 cm, and tumors greater than 10 cm. The mortality rate from SEF is high, with 25-57% of patients dying from the disease [6,7]. In the largest case series of 45 patients by Warmke et al., 42% of patients with SEF were alive with the disease at a median of 41 months, and 42% of patients were dead of the disease at a median of 49 months [25]. Only 11% of patients had no evidence of disease at a median of 63 months. The aggressive clinical course, albeit over a long time course, is a defining feature of SEF.

## Conclusions

SEF is a unique and rare malignancy typically of the deep musculature of the trunk and extremities. It is unusual in its low histological grade and indolent yet aggressive course, with late recurrences and high mortality. Aggressive surgical resection possibly combined with radiation therapy can be used for localized disease. Effective treatments for advanced or metastatic disease are limited. This case report demonstrates that a combination CLTA-4 and PD-1 inhibition may be an effective therapy. Larger studies to investigate possible therapeutic interventions like immunotherapy, based on a growing understanding of the molecular underpinnings of SEF, are warranted.
